# S-sulfhydration as a cellular redox regulation

**DOI:** 10.1042/BSR20150147

**Published:** 2016-03-03

**Authors:** Małgorzata Iciek, Danuta Kowalczyk-Pachel, Anna Bilska-Wilkosz, Inga Kwiecień, Magdalena Górny, Lidia Włodek

**Affiliations:** * Chair of Medical Biochemistry, Jagiellonian University, Medical College, 7, Kopernika Str., 31-034 Kraków, Poland; † Chair and Department of Pharmaceutical Botany, Jagiellonian University, Medical College, 9, Medyczna Str., 30-688 Kraków, Poland

**Keywords:** hydrogen sulfide, hydropersulfides, reactive sulfur species, S-sulfhydration, sulfane sulfur, sulfurtransferases

## Abstract

For many years reactive oxygen and nitrogen species (ROS and RNS) have been recognized as key messengers in the process of thiol-based redox regulation. Relatively recently, literature reports began to mention reactive sulfur species (RSS) and their role in thiol regulation. This review is focused on biogenesis and biological properties of RSS, including: hydropersulfides, polysulfides and hydrogen sulfide (H_2_S). Based on the most up-to-date literature data, the paper presents biological significance of S-sulfhydration process. In this reaction, sulfane sulfur is transferred to the–SH groups forming hydropersulfides. Protein cysteine residues, called ‘redox switches’ are susceptible to such reversible modifications. In line with the most recent reports, it was emphasized that sulfane sulfur-containing compounds (mainly hydrogen persulfides and polysulfides) are real and better mediators of S-sulfhydration-based signalling than H_2_S. We also overviewed proteins participating in the formation and transport of RSS and in mitochondrial H_2_S oxidation. In addition, we reviewed many reports about proteins unrelated to sulfur metabolism which are modified by S-sulfhydration that influences their catalytic activity. We also addressed the problem of the regulatory function of S-sulfhydration reaction in the activation of K_ATP_ channels (vasorelaxant) and transcription factors (e.g. NF_κ_B) as well as in the mechanism of therapeutic action of garlic-derived sulfur compounds. Some aspects of comparison between RNS and RSS are also discussed in this review.

## Abstract (precis)

This review is focused on formation and biological significance of hydropersulfides, i.e. S-sulfhydration process. Biogenesis and properties of reactive sulfur species and their role in redox signaling are presented. The effect of S-sulfhydration on protein function is discussed.

## INTRODUCTION

Reactive oxygen species (ROS), life threatening products of incomplete oxygen reduction, which damage biomolecules and impair their biological action, are an inevitable consequence of the appearance of oxygen and obligate aerobic organisms on the Earth billions of years ago. Thus, aerobic organisms had to develop a perfect antioxidant defence but simultaneously, they accomplished something more astonishing, namely, they used ROS at low concentrations as regulators of a variety of biological processes. Currently, it is known that the physiological level of oxidants performs regulatory function whereas their high levels cause oxidative stress and cell damage which leads to pathological states. The commonly accepted key messengers of redox signal transduction in the cell include not only reactive oxygen species (ROS) [1,2] but also reactive nitrogen species (RNS) [3] and reactive sulfur species (RSS) [4,5], which are the principal subject of this review.

### Reactive sulfur species

The concept of RSS has been postulated in 2001 by Giles et al. [6]. These authors proposed RSS as a group of redox-active molecules, which are formed *in vivo* under conditions of oxidative stress and can act as aggressive oxidizing agents [6]. According to this concept, RSS which are produced under oxidative stress, include thiyl radicals (RS^•^), sulfenic acids (RSOH), disulfides (RSSR), thiosulfinate (RS(O)SR), thiosulfonate (RS(O)_2_SR) and S-nitrosothiols (SNT). More recently, this definition was expanded to include sulfur-containing molecules, which are formed in physiological, non-oxidative conditions [7,8]. This hypothesis includes also another class of RSS, i.e. the products of cysteine transformations: hydrogen sulfide (H_2_S) and sulfane sulfur-containing compounds. In the literature, RSS created under physiological conditions (without oxidative stress) are called ‘the first class of RSS’ whereas ‘the second class of RSS’ means species formed upon the initial action of oxidative stress [7]. However, considering chronology of their appearance, reverse names would be more adequate. In this review, we will discuss processes mediated by RSS created during cysteine transformation, mainly H_2_S and products of its oxidation–inorganic polysulfides (H_2_S*_n_*) as well as hydropersulfides (RSSH).

**Figure 1:**
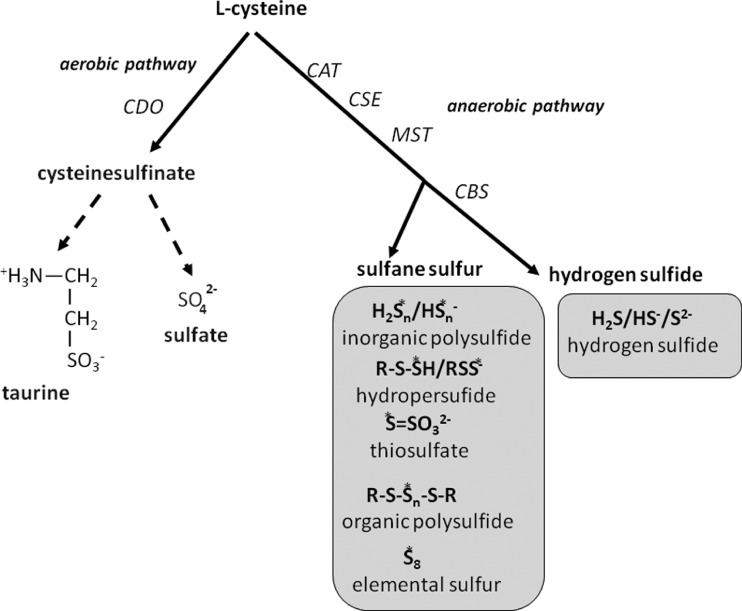
Aerobic and anaerobic transformation of L-cysteine Reactive sulfur species (RSS) formed in physiological, non-oxidative conditions are marked in grey boxes (S*; sulfane sulfur atom).

Cysteine sulfur can be metabolized via two main pathways: anaerobic route leading to reduced RSS (sulfane sulfur-containing compounds and hydrogen sulfide) and aerobic route yielding sulfates and taurine (Figure 1). In rats, almost one third of the total cysteine sulfur is catabolized to the reduced sulfur compounds whereas two thirds are oxidized via cysteinyl sulfinate to sulfates and taurine [9]. At present, it is not known which mechanisms underlie the vital decision in the cell about the rate of either the former or the latter process in cysteine metabolism.

#### Sulfane sulfur–definition, formation and importance

Sulfane sulfur-containing compounds contain a reactive sulfur atom occurring in 0 and −1 oxidation state and bound with another sulfur atom. The following examples of biologically active sulfane-sulfur-containing compounds are worth mentioning: RSSH and hydrogen persulfide (HSSH), thiosulfate (S_2_O_3_^2−^), organic (RS*_n_*R) as well as inorganic (H_2_S*_n_*) polysulfides (*n* ≥ 3) and elemental sulfur (S_8_) (Figure 1). Sulfane sulfur shows high activity in biological systems although protein carriers which stabilize and transport it are widespread [10,11]. Initially, it was thought that sulfane sulfur plays only a role in detoxification of cyanide originating from cyanogenic plants (forming thiocyanate) catalysed by rhodanese (Rhd), however, this view was substantially changed over the course of time. According to current knowledge, the sulfane sulfur-containing compounds, in particular RSSH and polysulfides (H_2_S*_n_* and RS*_n_*R) are very important for signalling, redox homoeostasis, regulation of metabolism and antioxidant defense in the cell [10–12]. Besides the beneficial effects of high activity of some RSS, especially organic polysul-fides, their highest concentrations can exert toxic effects that are connected with their ability to generate ROS [13].

**Figure 2:**
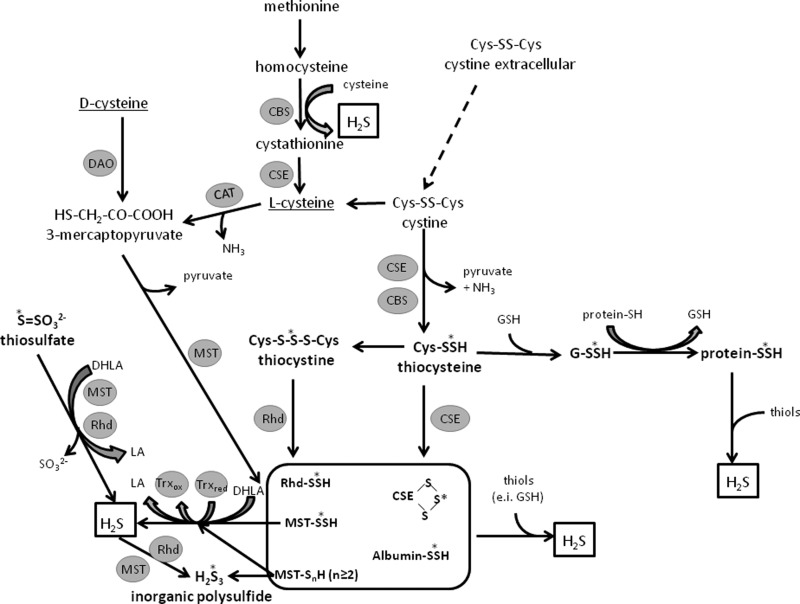
Generation and transport of sulfane sulfur as well as H_2_S production from L- and D-cysteine Sulfane sulfur and H_2_S are produced in reactions catalyzed by CAT, MST, CSE, CBS, Rhd and DAO. Sulfane sulfur-containing compounds are marked in bold. LA, lipoic acid.

Highly metabolically reactive sulfane sulfur-containing compounds are endogenous metabolites created in mammalian cells during anaerobic cysteine catabolic pathway in the reactions catalysed by such enzymes as cystathionine γ-lyase (CSE), 3-mercaptopyruvate sulfurtransferase (MST) as well as cysteine aminotransferase (CAT). The anaerobic pathway of L-cysteine conversion can be initiated by CAT-catalysed transamination of L-cysteine to 3-mercaptopyruvate (Figure 2). The latter compound is produced also in some tissues from D-cysteine by D-amino acid oxidase (DAO) [14]. 3-Mercaptopyruvate can play the role of a sulfur donor for different nucleofilic acceptors, including protein sulfhydryl groups with hydropersulfide formation. The sulfur atom is transported from 3-mercaptopyruvate by MST to form MST hydropersulfide (MST-SSH). The level of hydropersulfides, which contain sulfane sulfur, in the cells showing MST and CAT expression is twice higher compared with the cells lacking these enzymes [15]. Moreover, recent studies of Kimura et al. suggest the possibility of inorganic polysulfide production from 3-mercaptopyruvate. These studies performed in the mouse brain revealed the presence of H_2_S_3_ in the cytosol of brain cells and identified MST as the enzyme responsible for its production [16]. The experiments reported in that paper were performed without reducing agents, which could reduce H_2_S*_n_* to produce H_2_S. The presence of an acceptor or reducing agent (i.e. mercaptoethanol) can remove H_2_S from the active site of MST before H_2_S_3_ formation is complete. On the other hand, production of H_2_S_3_ was strongly suppressed in the presence of a high concentration of substrate (2 mM 3-mercaptopyruvate), because in the absence of reducing agents, excess of H_2_S_3_ in the active site of MST may suppress the progress of reaction, what was shown previously [17]. H_2_S_3_ identified by Kimura et al. was also produced from H_2_S by MST and rhodanese (Rhd) [16].

Another pathway of sulfane sulfur formation is associated with cystine conversion into cysteine persulfide called thiocysteine (Cys-SSH) by pyridoxal phosphate (PLP)-dependent CSE and cystathionine-β-synthase (CBS) (Figure 2). The production of thiocysteine from cystine was reported first time by Cavallini et al. [18]. Then, Szczepkowski and Wood demonstrated that thiocysteine could be converted to the sulfane sulfur-containing trisulfide, thiocystine which is a substrate for Rhd [19] (Figure 2). The detectable cystine concentration in cells is relatively low compared with the extracellular space. This suggests that in the cytosol where CSE and CBS are active, cystine is quickly transformed into thiocysteine. Thiocysteine concentration in cells was estimated at 1–4 μM [5].

It can be concluded that CSE and MST take part in sulfane sulfur formation and transport. In turn, Rhd only transports reacti-ve sulfur atom from donors (i.e. hydropersulfides, trisulfides) to acceptors (other sulfhydryl groups, cyanide). Not only enzymes involved in sulfur metabolism, like CSE, MST, Rhd but also proteins unrelated to sulfur metabolism, such as plasma albumin have the ability to bind and transport sulfane sulfur (Figure 2) [10].

#### Hydrogen sulfide as a gasotransmitter

The interest in RSS has arisen when hydrogen sulfide (H_2_S) has emerged as a signalling molecule. It happened much later after the signalling role of nitric oxide (NO) and carbon monoxide (CO) had been revealed. Most probably it was related to the conviction that H_2_S was a strong toxin which was upheld till the first reports of the physiological role of this gaseous molecule [20]. Since then reports on diverse biological activities of H_2_S have exploded. Hydrogen sulfide was shown to influence blood pressure, cell proliferation and apoptosis, angiogenesis, inflammatory processes, it was found to be cardioprotective, neuromodulatory and protective against hypoxia, which was described in many review articles [21–24]. These multidirectional physiological actions of H_2_S from the beginning have suggested its implication in diverse signalling processes. However, in spite of those numerous reports highlighting its multitudinous physiological effects, the mechanisms of its actions still have not been fully elucidated.

H_2_S is produced mainly from L-cysteine, however, recently a novel pathway of its production from D-cysteine catalysed by DAO has been discovered [14]. The enzymes involved in H_2_S production include PLP-dependent CSE and CBS as well as PLP-independent MST (Figure 2). The expression of the genes for these enzymes is tissue specific and CSE is the major H_2_S-producing enzyme in the cardiovascular system, liver and kidney [22]. H_2_S is formed in the reaction catalysed by CBS or CSE or non-enzymatically by reduction in hydropersulfides (Figure 2). MST produces H_2_S from 3-mercaptopyruvate, which is gene-rated from L- or D-cysteine. The sulfur atom is transported from 3-mercaptopyruvate by MST in the form of MST-SSH. It was shown that H_2_S could be released in the presence of dithiols [dihydrolipoic acid (DHLA), DTT, thioredoxin (Trx)]; however, in the presence of monothiols (GSH, cysteine) no release of H_2_S was observed [25]. It could probably be caused by sulfane sulfur transfer from MST-SSH to cysteine or GSH with the formation of Cys-SSH or GSSH. Moreover, sulfur atom of 3-mercaptopyruvate can be transferred to MST forming not only MST-SSH, but also higher forms of protein polysulfide MST-SS*_n_*H, from which H_2_S is released in the presence of reducing agents. MST, like Rhd, may also catalyse the reaction of H_2_S production from thiosulfate.

H_2_S differs from other gasotransmitters in its ability to dissociate. Its p*K*_a_ is 6.77 and in physiological conditions, i.e. at pH 7.4 in aqueous solutions, 80% of hydrogen sulfide is disso-ciated to HS^−^ anion and only 20% remains undissociated although S^2−^ concentration is extremely low. It is usually assumed that the hydrogen sulfide pool comprises H_2_S, HS^−^ and S^2−^. The concentration of H_2_S in human plasma and tissues is still controversial. Initially, reports indicated a high concentration of H_2_S in tissues, estimated at 10–100 μM and even 300 μM using methods available at that time [26,27]. Later studies revealed much lower concentrations (only 15 nM according to Furne et al.) and suggested that high estimates occurred most often to be artefacts [28]. This discrepancy can be explained by many various factors. First of all, there is a very close relationship between H_2_S and sulfane sulfur-compounds, which are regarded as a large H_2_S reservoir in biological conditions. H_2_S can be easily released from sulfane sulfur compounds in the presence of reductants (i.e. GSH) or by acidification required in some methods of H_2_S assay. This pool of RSS (H_2_S and sulfane sulfur) is tightly regulated in a dynamic manner (Figure 2). Moreover, most of the me-thods require using the standard (most often Na_2_S) and all tested reagents of various origin contained significant amount of inorganic polysulfides. Na_2_S shows a high vulnerability to oxidation with atmospheric oxygen, which depends also on the presence of metal ions and pH [29]. To obtain a stable stock solution, some authors have proposed washing the surface of Na_2_S crystals in redistilled argon-saturated water before their dissolution [30]. To be able to determine an actual H_2_S level, various new methods of H_2_S detection have been developed. Zhang et al. reported 0.5 μM concentration of free H_2_S in human plasma using naphthalimide-azide method with fluorescence detection [31]. Similar free H_2_S concentrations (below 1 μM) were obtained using the monobromobimane method coupled with RP-HPLC by other authors, who also assayed different forms of sulfane sulfur-containing compounds [32]. The obtained results clearly indicate that the free H_2_S level in biological systems is much lower that the level of sulfane sulfur-containing inorganic polysulfides. It seems that other RSS occurring in tissues at higher concentrations than H_2_S were responsible for the observed biological effects. It was confirmed by studies in which H_2_S*_n_* present in the brain activated the transient receptor potential ankyrin 1 (TRPA1) channels approximately 300 times more potently than H_2_S did [33]. In the light of these findings, small amounts of H_2_S*_n_* even in the presence of excess of H_2_S can serve as important biological regulators.

Toohey [34] has indicated that H_2_S is rather a biodegradation byproduct of sulfane-sulfur-containing compounds. S-sulfhydration reaction with H_2_S or H_2_S*_n_* participation plays a crucial regulatory role and at present researches' interest is focused on the biogenesis of RSS from endogenous and exogenous precursors and on regulatory properties of this reaction.

### Thiol-based redox regulation

Thiol-based redox regulation utilizes a characteristic feature of some redox-sensitive cysteine–SH groups in proteins, called ‘switches’ or ‘sensors’, which have a unique ability of cau-sing versatile, reversible oxidation to disulfides, sulfenic acids, S-nitrosothiols, mixed disulfides with proteins and hydropersulfides [35–38]. Protein–SH groups can also conjugate with aldehydes forming semiacetals, and can react with different electrophilic groups and metal cations. This capability of diverse modifications of protein–SH groups helps to grasp the mechanism and signifi-cance of–SH groups in signalling processes. However, it should be noted that not all protein–SH groups are comparably sensi-tive to such modification. Sensitivity of different protein cysteine–SH groups to oxidation depends mainly on the p*K*_a_ value, i.e. on the ability of its dissociation to thiolate anion (−S^−^), since thiolates are much stronger nucleophiles than thiols. In turn, thiol ionization is facilitated by local environment (positively charged neighbouring amino acids, hydrogen bonding and N-terminal α helix). Moreover, some proteins were observed to possess the specialized active-site architecture surrounding cysteine residue that activates substrate binding. For example, H-bonding interactions in peroxiredoxins, besides lowering p*K*_a_, activate the substrate [39,40]. On the other hand some studies revealed that besides low p*K*_a_, there were other factors influencing the thiol reactivity.

Kinetic studies reviewed and discussed by Winterbourn and Hampton distinctly indicated that in many cases protein thiols exhibited huge differences in reactivity to an oxidant despite similar p*K*_a_ values [37]. Numerous studies demonstrated that only some thiol proteins were oxidized under given conditions and that sensitivity to oxidation depended on an oxidizing agent. Moreover, the compartment where the oxidants (ROS, RNS or RSS) are generated is an important factor of response to oxidative stress due to the possibility of diffusion across the membrane and the distance of the sensitive–SH groups [37,38]. The selected redox-sensitive thiol proteins (sensors, switches), once oxidized, can facilitate the oxidation of other proteins which are implicated in the functional regulation of cell response to redox potential-related environmental changes [36]. When redox-active protein cysteine groups are localized in the active centre of key regulatory enzymes, then the redox modulation can control whole metabolic pathways. Some protein–SH groups can undergo reversible oxi-dation leading to regulatory allosteric effects.

Protein–SH groups can be oxidized not only by one-electron oxidants, i.e. radical species, like ROS, RNS and RSS but also by two-electron oxidants which can show a regulatory effect already at physiological concentrations, that was specifically emphasized by Jones [41,42]. These reversibly oxidized–SH groups are under control of glutathione (GSH), cysteine (Cys) and Trx, called ‘nodes’ and play an important role in signalling processes. The main function of the ‘nodes’ in redox regulation is to reduce the reversibly oxidized protein thiols, i.e. disulfides, sulfenic acids and S-nitrosothiols back to thiols. Trx is believed to be the main system reducing sulfenic acid although glutaredoxin (Grx) reduces mixed disulfides with proteins [42].

## BIOGENESIS AND BIOLOGICAL PROPERTIES OF HYDROPERSULFIDES, S-SULFHYDRATION PRODUCTS

Since the time when hydrogen sulfide (H_2_S) has been recognized to be a gasotransmitter, studies have concentrated not only on biological properties of ROS and RNS but also on RSS generated during anaerobic cysteine metabolism. Currently, the greatest interest in this group is placed on hydro-persulfides (RSSH and HSSH) and related inorganic and organic polysulfides (RS*_n_*R and H_2_S*_n_*, *n* ≥ 3), and H_2_S, due to their regulatory functions. Metabolism of sulfur amino acids leading to the formation of the above RSS is presented in Figure 2.

It appears that biological actions initially attributed to H_2_S are actually caused by sulfane sulfur-containing compounds. The formation of RSSH, in which sulfur is in −1 oxidation state, is one of possible reactions leading to reversible oxidation of thiol–SH groups (in which sulfur is in −2 oxidation state). Thus, protein S-sulfhydration to hydropersulfides is the oxidation reaction and is believed to be a fundamental process in thiol-based redox regulation which is confirmed by the constantly increasing number of reports of proteins regulated by this process [43–47]. In this review, we focus on thorough characterization of the protein S-sulfhydration reactions and on the significance of this covalent modification of–SH groups in thiol-based redox regulation. Hydropersulfides first drew attention of researchers when they were noticed to act as intermediates in the process of sulfur introduction during biosynthesis of vitamins, cofactors, thionucleosides and iron–sulfur proteins [48]. It indicates that apart from the regulatory function, cysteine-derived sulfane sulfur also participates in biosynthesis of key molecules in many biochemical processes.

It is worth mentioning that there are significant differences between properties of–SH and–SSH groups, namely, hydropersulfides (–SSH) show much greater chemical reactivity compared with the corresponding thiols (–SH) due to the increased nucleophilicity. At physiological pH, the acid–base balance of hydro-persulfides is shifted towards persulfide anion which means that hydropersulfides are stronger acids (lower p*K*_a_ value) and better hydrogen donors than their thiol counterparts [43]. Another significant difference is associated with much lower dissociation energy of the S–H bond in RSSH which equals 70 kcal/mol whereas the corresponding value for the S–H bond in RSH amounts to 92 kcal/mol [49]. It can be attributed to the increased stability of perthiyl radicals (RSS^•^) which are stabilized by resonance compared with thiyl radicals (RSS^•^). All these features make hydropersulfides very efficient antioxidants. For instance, a remarkable increase in the reducing capacity of GSSH relative to GSH was observed [5]. Toohey and Cooper [50] attributes this fact to the possibility of GSSH tautomerization to form thiosulfoxide, which easily donates electrons (reaction 1).

1 $$2\mathrm{GSSH}\to 2G\overset{\overset{S}{\left|\right|}}{S}H\to 2e^{-}+2H^{+}+\mathrm{GSS}\text{-}\mathrm{SSG}$$

A recent study by Ida et al. confirmed that only GSSH (gene-rated from GSSSG by glutathione reductase (GR)) could scavenge a marked amount of H_2_O_2_, whereas comparable amounts of GSH (formed in the reaction of GR with GSSG) produced no measurable H_2_O_2_ reduction [5]. It confirms that more nucleofilic hydropersulfides are superior reducing agents compared with their thiol counterparts. Moreover, these findings show that the sulfane sulfur in hydropersulfides is an extremely reactive reductant and is a potent antioxidant in cells. These authors underline that compared with GSH and H_2_S, hydropersulfides (mainly GSSH and Cys-SSH as well as protein hydropersulfides) are more efficient nucleophiles and reductants which fulfil critical regulatory functions in redox signalling in the cell [5].

Hydropersulfides are unstable compounds and in the presence of thiols can undergo desulfhydration accompanied by H_2_S release or dysproportiation reaction yielding thiol and elemental sulfur S_8_ (designated also as S^0^) [51] (reactions 2 and 3).

2 $$\;\mathrm{RSSH}\overset{R^{\prime }\mathrm{SH}}{\to }{\mathrm{RSSR}}^{\prime }\;+H_{2}S$$

3 $$\mathrm{RSSH}⟶\;\mathrm{RSH}+S^{0}$$

For this reason, sulfhydration can be also perceived as a storage method for the toxic H_2_S and as a path for elemental sulfur (S_8_) release [52]. The fact that H_2_S is released from hydro-persulfides in the presence of thiols explains why initially many biological functions were attributed to H_2_S. Numerous data from experimental studies indicate that rather not H_2_S but other reactive sulfur metabolites, mainly hydropersulfides or inorganic polysulfides, are capable of producing antioxidant and regulatory effects. What is more, it is increasingly more often emphasized that biological activity of H_2_S is mostly connected with the for-mation of hydropersulfides. Ida [5] and Toohey [34] even believe that this is not H_2_S but sulfane sulfur in a wider sense that fulfils an essential role in thiol-based redox regulation. However, many studies continue to report biological effects of S-sulfhydration by the H_2_S. Probably it results from the fact that, as mentioned earlier, all commercial reagents (Na_2_S or NaHS) contain signifi-cant amounts of sulfane sulfur. Results of Greiner et al. revealed the inhibition of lipid phosphatase (PTEN) activity by reversible oxidation of this enzyme by all tested H_2_S donors (i.e. Na_2_S or NaHS from various companies as well as the polysulfide K_2_S*_x_*), and demonstrated that the degree of inhibition depended on the sulfane sulfur content [53]. The use of the ‘purest’ preparation resulted only in a slight lowering of the activity (by less than 10%), whereas commercial preparations containing a considerable amount of sulfane sulfur caused even 60% inhibition [53].

Quantitative data indicate that hydropersulfides and inorganic polysulfides are widespread in cells and tissues and occur at much higher physiological concentrations than ROS or RNS. This confirms a crucial role of S-sulfhydration reaction in regulatory processes [5,45], and this is why this process and its biological significance attract increasing interest.

### Hydropersulfide formation

The hydropersulfides (RSSH) are formed by the oxidation of thiols (from −2 to −1 oxidation state). It is surprising, as also noticed by Greiner et al. [53], that many reports do not take notice of the fact that sulfur in H_2_S and in HS^−^ can act only as a reductant, and its direct participation in oxidation reactions of–SH groups to hydropersulfides (RSSH) is impossible. Therefore, hydropersulfides cannot be formed in a direct reaction of H_2_S with protein–SH groups. Hence, the S-sulfhydration reaction can occur only when one of the reagents (–SH group or H_2_S) is in the oxidized form.

The cysteine group can be S-sulfhydrated by HS^−^, when its–SH group is oxidized to sulfenic acid, disulfide, mixed disulfide or nitrosothiol [54,55]. The S-sulfhydration process of proteins consisting in the nucleophilic attack of HS^−^ anion on the reversibly oxidized–SH groups is presented in reactions 4–7. In these reactions, the sulfur of hydrosulfide anion is transformed into sulfane sulfur present in the generated hydropersulfide.

4
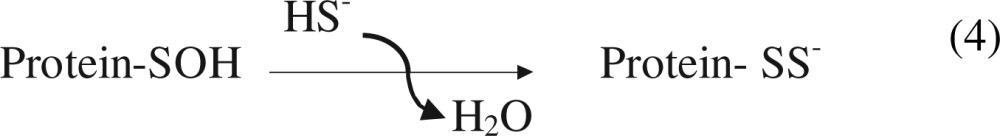


5
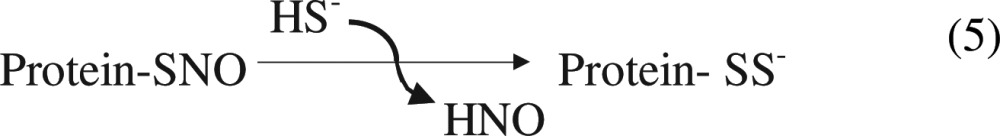


6
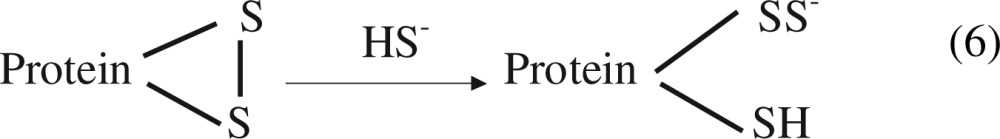


7
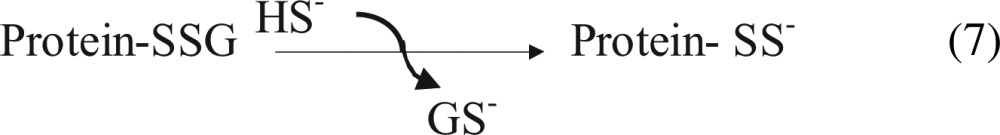


It appears that in cytosolic environment which is reducing due to the GSH system, the protein–SH groups are rarely oxidized, thus this mechanism of the sulfhydration will not be preferred. However, when GSH is deficient or in more oxidizing environment, like mitochondria or endoplasmic reticulum those reactions can occur. The formation of hydropersulfide of sulfide quinone oxidoreductase (SQR) during mitochondrial H_2_S oxidation is an example of the S-sulfhydration reaction via the above mechanism (see Hydrogen sulfide oxidation pathway: hydropersulfides as the intermediates section). Recently, Vasas et al. [56] performed kinetic studies on disulfide reduction reaction by HS^−^. Those authors demonstrated that the reaction occurred via multistep equilibria through disulfide, hydropersulfide and inorganic polysulfides (reactions 8–10). They suggested that reduction in disulfide (RSSR) by HS^−^ was a highly system-specific process, and thermodynamic and kinetic premises indicated a tight regulation. Those authors proposed that chemical nature of the disulfide moiety would play a major role in the feasibility of its reduction by hydrosulfide anion [56]. Although those studies were conducted on non-protein disulfides (DTNB, CysSSCys, GSSG), it appears that the results can be applied to protein disulfides. In proteins, the reaction will depend on the surrounding amino acids. The presented mechanism indicates that inorganic polysulfides (HS*_n_*^−^) are formed in parallel with hydropersulfides (RSS^−^).

8 $$\mathrm{RSSR}+HS^{-}⟶\;\mathrm{RS}S^{-}+\mathrm{RSH}$$

9 $$\mathrm{RS}S^{-}+HS^{-}⟶\;RS^{-}+\mathrm{HS}S^{-}$$

10 $$n\mathrm{HS}S^{-}+HS^{-}⟶\;H{S_{\left(n+1\right)}}^{-}+\left(n-1\right)HS^{-}$$

Recently, Cuevasanta et al. [57] in their excellent work stu-died the reactivity of HS^−^ towards symmetric disulfides (RSSR) and mixed disulfides with albumin (HSA). They observed the 20-fold increase in HSA persulfide reactivity in comparison with the HSA thiol at pH 7.4. It shows a very improved nucleophilicity of persulfides with respect to the parent thiols and H_2_S. Experiments with cells in culture indicate that hydropersulfide formation increases upon exposure to hydrogen peroxide. These authors presented also kinetic evidence that the hydropersulfides have much better nucleophilic reactivity due to alpha effect [57].

S-sulfhydration reaction can also occur via reaction of protein–SH group with oxidized forms of hydrogen sulfide (HSOH or HSSH) (reactions 11 and 12).

11
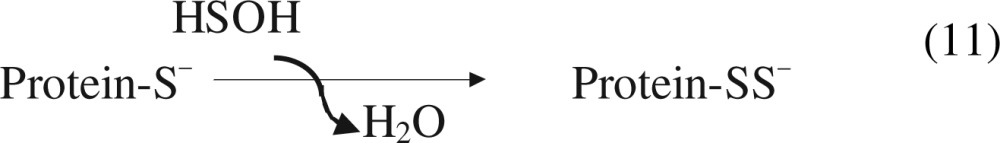



12
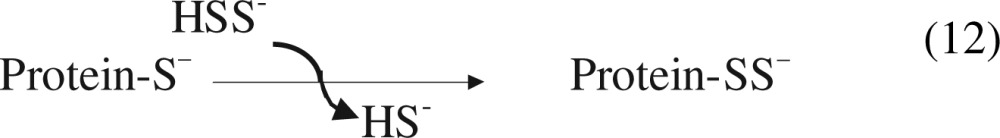



Nagy and Winterbourn [58] investigated the reaction of HS^−^ with hypochlorous acid (HOCl). This reaction leads initially to sulfenyl chloride which undergoes hydrolysis yielding sulfenic acid and then condensation with another HS^−^ anion to produce polyhydropersulfides (reaction 13). Products of this reaction can be oxidizers of protein–SH groups which leads to the formation of protein hydropersulfide (reaction 12).

13 $$\begin{array}{ccc} HS^{-} & \overset{\mathrm{HOC}1}{\to } & \mathrm{HS}\text{-}\mathrm{Cl}\overset{H_{2}O}{\to }\mathrm{HS}\text{-}\mathrm{OH}\overset{HS^{-}}{\to }\mathrm{HS}\text{-}S^{-}\\ & \overset{{(HS^{-})}_{n}}{\to } & H{\left(S\right)}_{n}S^{-}\;n=1-7 \end{array}$$

Moreover, as already mentioned, H_2_S can be easily oxidized to inorganic polysulfides, so sulfide and hydrosulfide salts are usually contaminated with sulfane sulfur. Thus, inorganic polysulfides can be intermediates in the–SH to–SSH transformation pathway and participate in the thiol-based redox signalling [53,59]. Sulfane sulfur present in polysulfides can be easily transferred to thiolate anion with hydropersulfide formation [59]. So, when we add sulfide solution to a protein solution, in fact we introduce the sulfane sulfur. Based on the above observations, it is not difficult to imagine that indeed inorganic polysulfides and hydropersulfides can be responsible for biological activity of H_2_S. Moreover, some of RSS containing sulfane sulfur (mainly hydropersulfide and thiosulfate) are generated during mitochondrial sulfide oxidation pathways (see Hydrogen sulfide oxidation pathway: hydropersulfides as the intermediates section) and in this way they can modify activity of target proteins.

Hydropersulfides can also be produced via the radical pathway [29]. One electron oxidation of H_2_S leads to HS^•^ formation, which can next react with protein thiols to give finally hydropersulfide and superoxide radical anion in accordance with reactions 14 and 15.

14 $$HS^{•}+\mathrm{Protein}\text{-}\mathrm{SH}⟶\;\mathrm{Protein}\text{-}\mathrm{SS}H^{-\cdot }+H^{+}$$

15 $$\mathrm{Protein}\text{-}\mathrm{SS}H^{-\cdot }+O_{2}⟶\;\mathrm{Protein}\text{-}\mathrm{SSH}+{O_{2}}^{-\cdot }$$

### Involvement of thiocysteine in the glutathione and protein S-sulfhydration process

Protein S-sulfhydration can occur also as the result of sulfane sulfur transfer from low molecular weight hydropersulfides to protein–SH groups. Thiocysteine, generated from cystine by CSE or CBS (reaction 16), can be involved in this S-sulfhydration process (Figure 2).

16 $$\mathrm{Cys}\text{-}S\text{-}S\text{-}\mathrm{Cys}\overset{\mathrm{CSE},\mathrm{CBS}}{\to }\mathrm{Cys}\text{-}SS^{*}H+\mathrm{pyruvate}+NH_{3}$$

Moreover, thiocysteine sulfane sulfur can be transferred to GSH, present in cells at high concentrations, with the formation of glutathione hydropersulfide (GSSH) (reaction 17) [5].

17
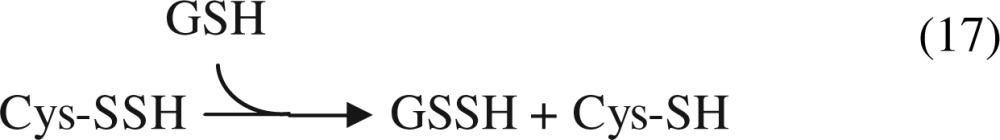


According to literature data, the GSSH concentration in mammalian tissues is high (over 100 μM in the brain and about 10–100 μM in other organs [5,54]), which justifies the conclusion that GSSH is the most widespread low molecular weight hydro-persulfide, playing a dominant role in S-sulfhydration reactions. However, it seems that the published physiological hydropersul-fide concentrations are only estimates because appropriate analy-tical standards are lacking due to exceptional instability of those compounds.

Ida et al. [5] demonstrated that GSSH could react with glutathione disulfide (GSSG) which led to the formation of the trisul-fide (GSSSG), and then in subsequent reactions even higher GSH polysulfides could be generated (reaction 18).

18
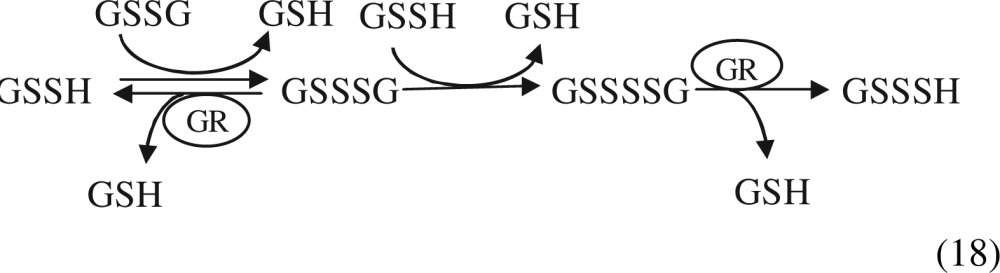


Those authors revealed that all GSH polysulfides (GSSG, GSSSG, GSSSSG) could be reduced in the reaction catalysed by GR leading to the generation of GSH-based forms of hydro-persulfides (GSSH, GSSSH, GSSSSH). The high GSSH content is maintained by CSE and CBS generating Cys-SSH and by GR activity that regenerates GSSH and GS(S)*_n_*H from oxidized GSH polysulfides in cells [5].

In the next steps, GSSH and other low molecular weight hydropersulfides can transfer sulfane sulfur to other–SH groups, most of all to the–SH groups of proteins [5,45,54]. The reactions leading to the exchange of–SH and–SSH groups, like the reaction of GSSG with protein–SH groups, are called S-transsulfhydration reactions. It indicates that the formation of protein hydropersulfides in cells can be a result of sulfane sulfur transfer from low-molecular-weight compounds (reaction 19) to a specific redox-sensitive cyteine residue in the target protein. Many data indicate that Cys-SSH and then GSSH are the main sources of sulfane sulfur for S-sulfhydration reactions of proteins despite the fact that there are reports of hydropersulfide formation in the reaction of HS^−^ anion with reversibly oxidized–SH groups.

19
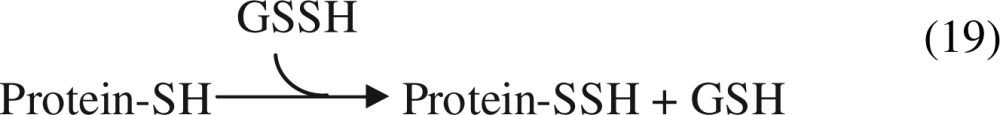



Sulfane sulfur present in protein hydropersulfides (Protein-SSH) can then be transferred to acceptor–SH groups of other proteins (transsulfhydration) (reaction 20) [5,54], which repre-sents the thiol-based regulatory mechanism.

20
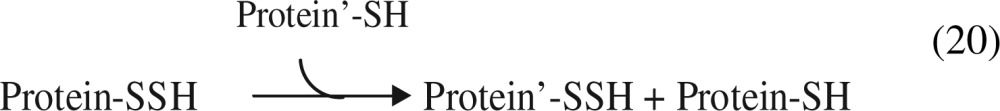



The protein hydropersulfide formation can prevent the accumulation of dangerous H_2_S excess, and provides a cellular regulatory mechanism of its level. It is commonly believed that protein hydropersulfides are a storage form of H_2_S, which can be released e.g. under the influence of thiols (reaction 21) (desulfhydration process) [60].

21
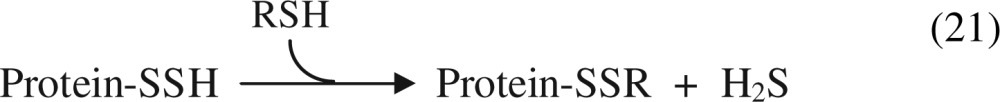



### Hydrogen sulfide oxidation pathway: hydropersulfides as the intermediates

Since interest in biological actions of hydropersulfides and hydrogen sulfide (H_2_S) is on the rise, it would be fundamental to know not only H_2_S biosynthesis but also biodegradation paths. Since H_2_S is an active molecule and in higher concentrations it is very toxic, its cellular level has to be tightly regulated because it is able to suppress the respiratory chain by inhibiting cytochrome oxidase. It means that an imbalance between H_2_S biogenesis, its storage capacity in the hydropersulfide form (–SSH) and biodegradation is dangerous. Thus, maintaining the physiological concentration of H_2_S in tissues is of crucial importance. H_2_S is biodegraded mostly in mitochondria through a series of oxidations via hydropersulfides, sulfite, thiosulfate and sulfate [60]. Mitochondrial H_2_S oxidation is catalysed by the following enzymes: sulfide quinone oxidoreductase (SQR) persulfide dioxygenase (ETHE1) and Rhd [61].

**Figure 3:**
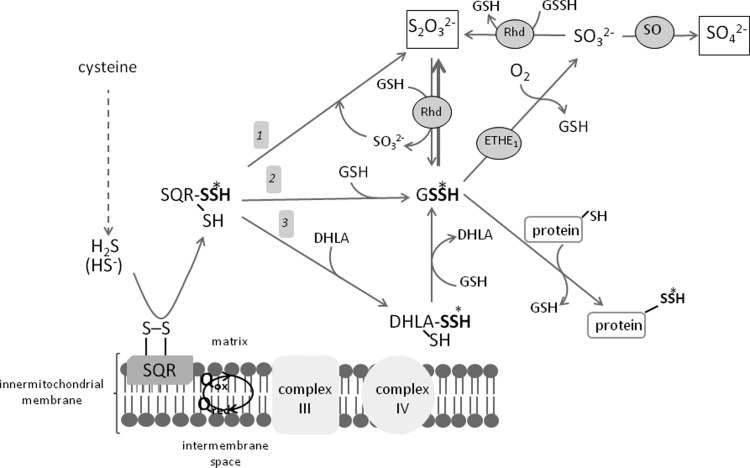
Formation of hydropersulfides during mitochondrial H_2_S oxidation Thiosulfate and sulfate are the main products of H_2_S oxidation. Enzymes participating in this process include: SQR, ETHE1, Rhd, SO.

The H_2_S degradation pathway begins with the reaction catalysed by SQR. This enzyme catalyses a two-electron oxidation of H_2_S to the sulfane sulfur-containing persulfide SQR-SSH [62,63]. This reaction is like a gate to the H_2_S oxidation pathway. In this reaction, ubiquinone is an electron acceptor, and this links H_2_S catabolism with oxidative phosphorylation and makes H_2_S the first inorganic substrate for electron transfer chain [64]. This indicates that mitochondrial H_2_S catabolism is conjugated with ATP biosynthesis. SQR is an inner mitochondrial membrane-bound flavoprotein belonging to the class of disulfide oxidoreductases. This enzyme is characterized by a low *K*_m_ value (μM) and a high catalytic sulfide turnover rate, supporting efficient catabolism of the toxic substrate [63]. A physiological acceptor of sulfane sulfur from SQR-SSH has not been identified unequivo-cally, yet. Some authors have postulated that human SQR utilizes sulfite as a persulfide acceptor yielding thiosulfate as a product, thus, this reaction is rapid and highly efficient at physiological pH (Figure 3, path 1) [62]. On the other hand, studies of Libiad et al. [60] have demonstrated that in addition to sulfite, GSH fun-ctions as a persulfide acceptor for human SQR leading to GSSH (Figure 3, path 2). It is not excluded that there are SQR persulfide acceptors other than sulfite and GSH (e.g. DHLA, Trx, cysteine), which after accepting sulfane sulfur can be reduced by GSH (Figure 3, path 3 presented for DHLA) [65]. Thus, depending on the route, GSSH is the next direct or indirect metabolite on the H_2_S oxidation pathway. It is the known substrate of ETHE1, the second enzyme participating in sulfide oxidation. ETHE1 is a non-heme iron-containing protein in mitochondrial matrix that catalyses the oxidation of the persulfide, consuming molecular oxygen [66,67]. Sulfite formed in the ETHE1-catalysed reaction can be oxidized to sulfate by sulfite oxidase (SO) or can accept persulfide sulfane sulfur yielding thiosulfate in the reaction catalysed by Rhd (Figure 3). An inherited mutation in persulfide dioxygenase gene results in an autosomal recessive disorder called ethylmalonic encephalopathy (EE). In patients suffering from deficient ETHE1 activity, the level of H_2_S and S_2_O_3_^2−^ rises whereas that of SO_3_^2−^ declines. Toxic sulfide concentrations cause the vascular endothelium damage and lead to neurological failure, chronic diarrhea and disturbances in mitochondrial energy metabolism [68]. Since the hydropersulfide GSSH is an ETHE1 substrate, this enzyme should be perceived not only as a H_2_S biodegradation tool but also as an enzyme controlling the hydropersulfide level and thus regulating the degree of protein S-sulfhydration in cells.

As mentioned above, rhodanese is the third crucial protein in the H_2_S oxidation route. Rhd is a mitochondrial enzyme known for its ability to utilize sulfane sulfur in CN^−^ detoxification reactions with thiocyanate formation [69]. Another substrate of rhodanese, thiosulfate can be generated, for instance, by sulfane sulfur transfer from SQR-SSH to sulfite. Some authors have proposed that thiosulfate is the main substrate for glutathione-dependent Rhd (thiosulfate sulfurtransferase) [70]. This reaction produces GSH persulfide and regenerates sulfite. However, studies of Hildebrandt and Grieshaber [61] and Libiad et al. [60] have clearly indicated that Rhd preferentially synthesizes rather than utilizes thiosulfate. The *K*_m_ values obtained in those studies have strongly suggested that sulfite and persulfides rather than thiosulfate and cyanide are preferable natural substrates for Rhd.

The main products of H_2_S metabolism and forms of sulfur excreted from the body include thiosulfate and sulfate. In the liver, sulfate is a predominant end product of H_2_S metabolism in the presence of GSH, whereas in the absence of GSH, a mixture of sulfate and thiosulfate is generated [71]. Under conditions of high H_2_S levels, an elevated level of blood and urinary thiosulfate was observed [70]. Interestingly, the S-sulfhydration reaction occurs twice in the mitochondrial H_2_S oxidation process, namely du-ring the formation of the hydropersulfides SQR-SSH and GSSH. Sulfane sulfur belongs not only to the structure of hydropersul-fides but also to thiosulfate (SSO_3_^2−^) created in the Rhd-catalysed reactions. The whole process of H_2_S oxidation appears to follow a surprisingly tangled path. However, the seemingly complicated scheme of H_2_S oxidation is biologically justified. Recently, Mishanina et al. [8] in their very interesting paper posited that sulfide oxidation pathway should be considered not only as a mechanism for utilization of excess sulfide, but principally as the way for generation of RSS, including persulfides, polysulfides and thiosulfate.

Thiosulfate formation can be viewed as one of the methods of H_2_S storage in cells, from which it can be released by thio-sulfate reductase. The elegant experiments of Koj et al. [72] and Skarżyński et al. [73] support the central role for thiosulfate as a key intermediate in the H_2_S oxidation. Since, as commonly known, sulfate is the final product of aerobic cysteine sulfur metabolism, the thiosulfate concentration in body fluids and tissues is assumed to be an indicator of H_2_S production in the body.

**Figure 4:**
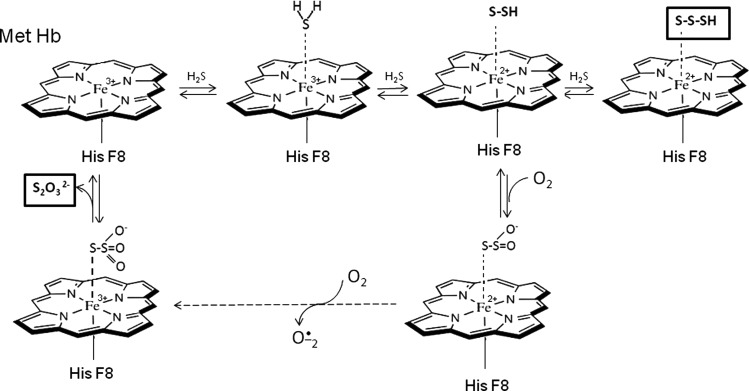
Scheme of MetHb-dependent H_2_S oxidation to thiosulfate and inorganic polysulfides bound to hem iron Modified from [74]: Vitvitsky, V., Yadav, P.K., Kurthen, A., Banerjee, R. (2015) Sulfide oxidation by a noncanonical pathway in red blood cells generates thiosulfate and polysulfides. J. Biol. Chem. **290**, 8310–8320.

H_2_S catabolism is tightly connected with mitochondrial re-spiratory chain, thus, it would be interesting to know how this compound is degraded by red blood cells lacking mitochondria. Recent studies of Vitvitsky et al. [74] discovered a novel mecha-nism of H_2_S oxidation in erythrocytes. They revealed that RBCs utilized methaemoglobin to catalyse H_2_S oxidation producing thiosulfate and polysulfide (Figure 4). The postulated mechanism explains how erythrocytes maintain low H_2_S levels in circulation, moreover, it cannot be excluded that additional hemoproteins might be involved in maintaining the H_2_S homeostasis..

### S-sulfhydration of proteins

Not only enzymes involved in sulfur metabolism, like CSE, MST or Rhd [10,11,75] but also proteins entirely unrelated to sulfur metabolism have the ability to bind and transfer sulfane sulfur in the form of hydropersulfides or trisulfides. They include, for instance, the above-mentioned plasma albumin which transports sulfane sulfur in the form of hydropersulfide [10]. The important role of albumin in sulfane sulfur transport was proven by the presence of sulfane sulfur in organs with no or trace enzymatic activity related to sulfane sulfur biosynthesis [76].

Numerous enzymes have been reported to be S-sulfhydrated *in vitro* and in intact cells, including actin, glyceraldehyde-3-phosphate dehydrogenase (GAPDH) [44], protein tyrosine phosphatase (PTP1B) [77], PTEN [53], Ni-containing carbon mono-xide dehydrogenase [78], Cu/Zn superoxide dismutase (SOD) [79], malate dehydrogenase [80] and 5-aminolevulinate synthase [81].

In these enzymes,–SH groups function as sensors (switches) able to bind sulfane sulfur and thus to regulate their activity by hydropersulfide formation. The S-sulfhydration in the active centre is also required for the activation of xanthine oxidase and aldehyde oxidase which was demonstrated already in the 1970s [82,83]. Studies on these enzymes suggested that sulfane sulfur occurring in the form of hydropersulfides was a natural component of these proteins. Removal of sulfane sulfur by cyanide treatment (cyanolysis reaction) inactivated those enzymes (reaction 22). The cyanolysis reaction is a proof of the presence of–SSH group in a protein and is a fundamental characteristic reaction of hydropersulfides and other forms of sulfane sulfur.

22 $$\mathrm{Enzyme}\text{-}SS^{-}+CN^{-}⟶\;\mathrm{Enzyme}\text{-}S^{-}+\mathrm{SC}N^{-}$$

Correspondingly, the incubation of malate dehydrogenase with thiosulfate and Rhd, i.e. an enzyme transforming–SH into–SSH groups, was shown to activate this enzyme (reaction 23) [80].

23 $$\mathrm{Enzyme}\text{-}S^{-}+Na_{2}S_{2}O_{3}\overset{\mathrm{Rhd}}{\to }\mathrm{Enzyme}\text{-}SS^{-}+\;Na_{2}SO_{3}$$

Studies of Mustafa et al. [44] revealed that 39 of the liver proteins are sulfhydrated under physiological conditions including GAPDH, β-tubulin and actin, and S-sulfhydration of these proteins can be reversed by DTT. Sulfhydration augments GAPDH activity and enhances actin polymerization. The S-sulfhydration reactions in those experiments were dependent on the H_2_S endogenous generation potential or its exogenous supplementation. Other studies showed that reaction of Cu/Zn SOD with sodium sulfide led to the formation of persulfide group at Cys^111^. This modification made the acid-induced denaturation of SOD fully irreversible [79]. Ni-containing carbon monoxide dehydrogenases from bacteria catalyse the reversible oxidation of CO to CO_2_ in the active site containing nickel, iron and sulfur. Studies of Kim et al. have revealed that the persulfide bond is essential for the stability and catalytic activity of the Ni–Fe–S clusters [78]. The protein tyrosine phosphatase B1 (PTP1B) is a member of the PTP family, which regulates various signal transduction pathways. This enzyme has the Cys residue in the active centre that is very susceptible to reversible oxidation. *In vitro* and *in vivo* studies demonstrated that PTP1B could be sulfhydrated in its active site which resulted in the inhibition of phosphatase activity. Inactivation of PTP1B by H_2_S (probably containing sulfane sulfur) was reversed preferentially by Trx [77]. Greiner and co-workers have studied oxidation of protein thiol groups via S-sulfhydration reaction, using PTEN as a protein model [53]. The activity of this enzyme strictly depended on the free thiol of Cys^124^ and oxida-tive modification of this–SH group led to PTEN inactivation. It was found that the addition of sulfane sulfur to cysteine in the active centre of PTEN resulted in the inhibition of the enzyme. When PTEN was treated with H_2_S in the presence of GSH, its inhibitory effect on PTEN activity was significantly weaker because GSH prevented formation of the hydropersulfide form in the PTEN active centre. Additionally, recent studies of Ohno et al. [84] revealed that both Cys^71^ and Cys^124^ in PTEN were the targets for S-sulfhydration. Further, the CBS knockdown in human neuroblastoma cells SH-SY5Y reduced this modification and made PTEN more sensitive to modification by NO.

The above examples showed that S-sulfhydration of enzymes affects their activity, which means that it has a regulatory cha-racter. Unlike other kinds of modifications of–SH groups (e.g. S-nitrosation, S-glutathionylation), S-sulfhydration often increases catalytic activity of proteins, however, studies on PTP1B and PTEN indicated that this modification could also suppress their activity.

In the above-mentioned studies, Greiner et al. evidenced that inorganic polysulfides, formed in NaHS solutions, were the oxidizing species responsible for PTEN S-sulfhydration. All tested ‘H_2_S donors’, like Na_2_S, gaseous H_2_S and GYY4137, led to polysulfide-mediated oxidation of PTEN via addition of sulfane sulfur atom to an important Cys residue in this protein [53]. Those and other studies clearly suggest that the effects previously attributed to H_2_S are rather mediated by inorganic polysulfides and other compounds containing sulfane sulfur. Actual documented low concentrations of H_2_S in tissues and higher levels of sulfane sulfur-containing compounds additionally confirm that the latter compounds play an essential role in the thiol-based redox regulation. However, other physiological actions of H_2_S cannot be excluded, like its interaction with NO and CO and metal ions, and interactions with hemoproteins, e.g. haemoglobin or cytochrome oxidase *c*.

#### Regulatory action of S-sulfhydration in the K_ATP_ channel activation process

ATP-sensitive potassium (K_ATP_) channels are multi-subunit protein complexes distributed on the surface of the cell and mitochondrial membranes of many different cell types. K_ATP_ channels are octameric complexes of two types of membrane-protein subunits. They are composed of four pore-forming Kir subunits and four regulatory subunits known as SUR (sulfonylurea receptor). Each Kir subunit associates with one SUR subunit [85]. Transmembrane Kir subunits allow for K^+^ ion influx into the channel complex, although SUR subunit plays a receptor role for different pharmacological compounds which activate or inhibit the channel opening [85]. A drop in the ATP level and a rise in cellular ADP level are physiological triggers of channel opening whereas sulfonylurea derivatives are pharmacological K_ATP_ channel openers.

**Figure 5:**
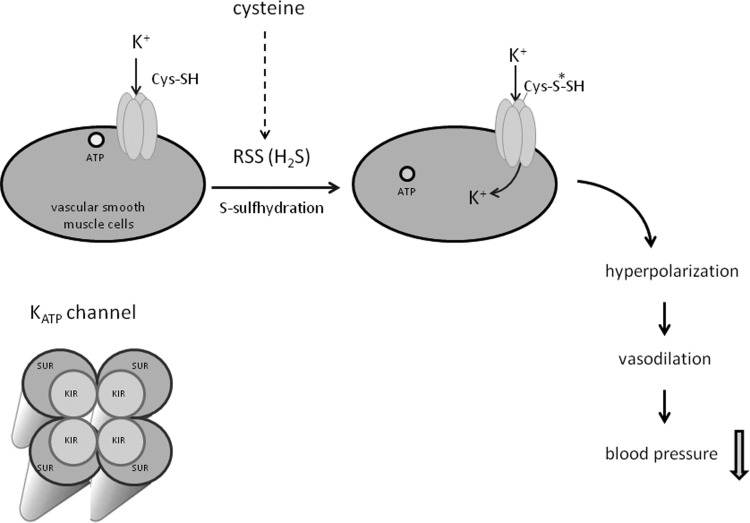
S-sulfhydration of K_ATP_ channel Formation of hydropersulfide form of K_ATP_ channel leads to its activation and vasodilatation. RSS, reactive sulfur species.

It is strongly suggested that K_ATP_ channels are one of the major targets of H_2_S and are activated via S-sulfhydration (Figure 5) [86,87]. All studies on the K_ATP_ channel S-sulfhydration suggest that this process is triggered by H_2_S, however, it should be remembered that these studies utilized Na_2_S or NaHS, which contain significant amounts of sulfane sulfur. To verify which RSS are actually responsible for the biological effects, it would be necessary to carry out comparative studies between the effect of Na_2_S and analogical amounts of inorganic polysulfides.

The activation of K_ATP_ channels in vascular smooth muscle cells involves the formation of hydropersulfide form of the protein, which reduces its ATP binding affinity and results in vasorelaxation [87]. Mustafa et al. [87] evidenced that vasorelaxant action of H_2_S was connected with sulfhydration at Cys^43^, i.e. one of nine cysteine residues in Kir6.1 subunit. On the other hand, some authors suggested that H_2_S sulfhydrated SUR2B subunit, not Kir6.1 subunit [88,89]. Irrespective of which K_ATP_ channel subunit is the main H_2_S target, hydropersulfide formation plays a crucial role in potassium channel activation. Moreover, some studies demonstrated that sulfhydration of SUR2B subunit modi-fied tyrosine nitration in Kir6.1 subunit, what suggests the interaction between these two posttranslational modifications [89].

Inhibition of potassium channel by the specific inhibitor glibenclamide blocks vasorelaxant action of H_2_S (or RSS) [87]. Likewise, the application of propargylglycine, an inhibitor of the H_2_S and sulfane sulfur synthesizing enzyme in vascular cells (i.e. CSE), blocks vasorelaxant action of H_2_S. The CSE knockout mice show vascular dysfunction and atherosclerosis which facilitates progression of coronary artery disease [90]. For this reason, the drugs increasing endogenous production of H_2_S and sulfane sulfur, like *N*-acetylcysteine, and H_2_S precursors can be beneficial in antihypertensive therapy.

#### S-sulfhydration reaction in the transcription factor NF_κ_B activation process

**Figure 6:**
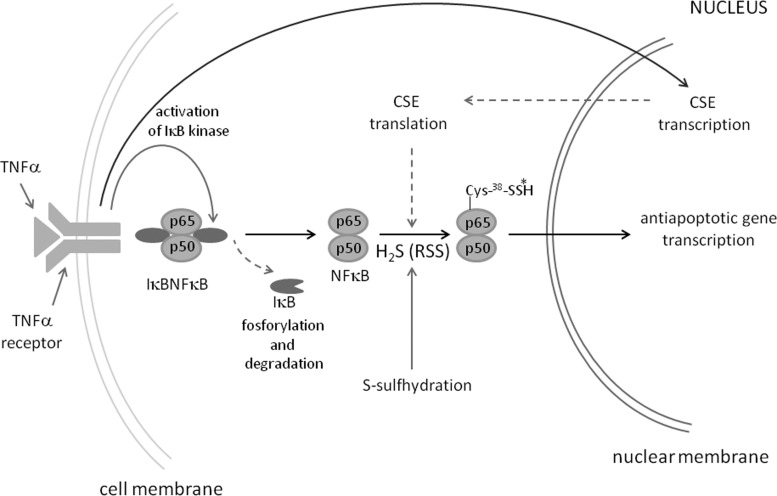
S-sulfhydration of the transcription factor NF_K_B and antiapoptotic gene transcription CSE, cystathionine-γ-lyase, RSS, reactive sulfur species. Modified from [43]: Paul, B.D. and Snyder, S.H. (2012) H_2_S signaling through protein sulfhydration and beyond. Nature Rev. Molecular Cell Biol. **13**, 499–507.

Signals from hormones, growth factors, cytokines and neurotransmitters are transduced in cells and transmitted to the transcription machinery in the nucleus by a class of proteins called transcription factors, and NF_κ_B is their representative. This anti-apoptotic factor is inactive under basal conditions because it is bound to the inhibitor I_κ_B (I_κ_BNF_κ_B). The multifunctional proinflammatory cytokine, tumour necrosis factor alpha (TNFα) activates I_κ_B kinase which leads to its phosphorylation and degradation and to the release of NF_κ_B and its translocation to the nucleus (Figure 6). Sen and co-workers showed that TNFα sti-mulated transcription of CSE and led to S-sulfhydration of NF_κ_B. H_2_S generated in CSE-catalysed reactions creates hydropersul-fide at Cys^38^ in the p65 subunit, which contributes to transcription of antiapoptotic proteins in the nucleus [91]. Therefore, TNFα-induced S-sulfhydration of p65 subunit of NF_κ_B improves survival of cells. Some studies showed a dramatic increase in H_2_S production in PLC/PRF/5 hepatoma cells compared with human LO2 hepatocyte cells. Moreover, treatment of these hepatoma cells with NaHS as a H_2_S donor, markedly increased CSE and CBS expression leading to NF_κ_B activation, decreasing the number of apoptotic cells and increasing cell viability [92].

### Capability of hydropersulfide, sulfane sulfur and hydrogen sulfide formation from garlic-derived sulfur compounds

Therapeutic potential of garlic has been known for a long time. Garlic is known to counteract atherosclerosis, reduce glucose and cholesterol level, strengthen the immune system, and to show anticancer, antioxidant and hypotensive activities [93–95]. The main organosulfur compound occurring in garlic called allicin is unstable and rapidly decomposes mostly to diallyl disulfide (DADS) and diallyl trisulfide (DATS). Benavides et al. [96] have demonstrated that garlic owes its vasoactive effect to the transformation of diallyl polysulfides to the sulfane sulfur-containing hydropersulfides, which react with thiols to release H_2_S.

The reaction of DATS, which originally has sulfane sulfur in its structure, with GSH produces the mix disulfide allylglutathione and the low molecular weight hydropersulfide allylperthiol (reaction 24), from which H_2_S is released in the reaction with GSH [96] (reaction 25).

24
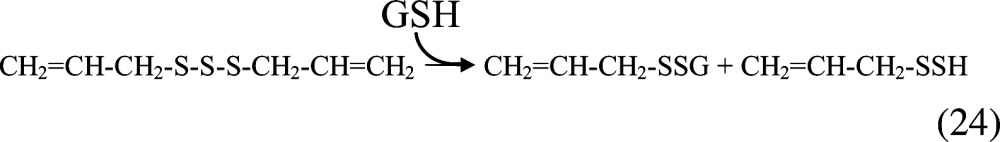



25
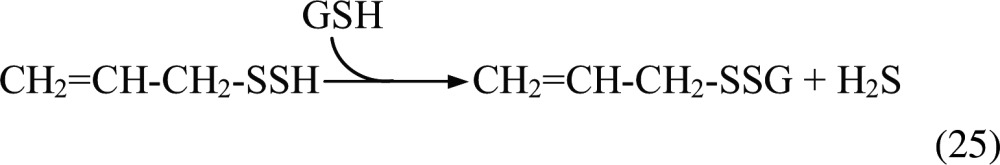



In turn, the reaction of DADS with GSH yields S-allylglutathione and allylperthiol (reaction 26), which reacts with GSH releasing H_2_S according to reaction 25 [96].

26
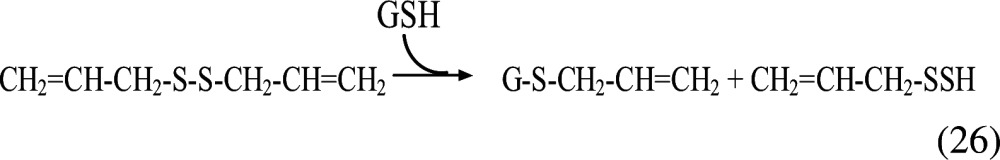



In the above reactions, protein thiols can compete in cells with GSH and can react with allylperthiol which may lead to covalent modification of proteins and formation of mixed disul-fides. Moreover, DATS can directly transfer reactive sulfane sulfur to protein–SH groups which generates protein hydropersulfides. Greiner et al. [53] showed that DATS efficiently inhibited PTEN activity by oxidation of its thiol group in HEK293T cells. On the other hand, DADS, which is a disulfide, can be a source of sulfane sulfur and can transfer this active sulfur atom in the tautomerization reaction to thiosulfoxide (reaction 27) [10].

27
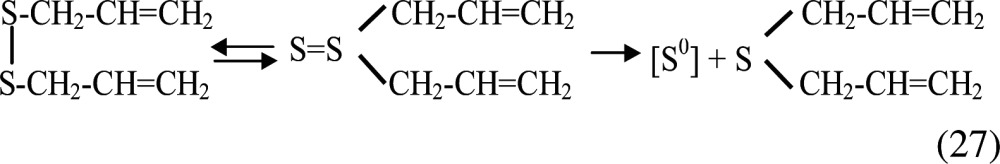



Hence, it seems that studies on biological and pharmacological activity of garlic are connected with hydropersulfides, sulfane sulfur and H_2_S.

## INTERCONNECTIONS AND COMPARISON BETWEEN ACTIVITIES OF REACTIVE NITROGEN SPECIES (RNS) AND REACTIVE SULFUR SPECIES (RSS)

Studies of recent years unravelled in greater depth the biogenesis and regulatory mechanisms governing ROS- and RNS-based responses to different stimulators. Our knowledge of RSS is much more patchy because these compounds, like H_2_S, attracted researchers' interest much later. Investigations of NADPH oxidase or NO synthase isoforms with different specific inhibitors contributed much to a better understanding of the regulatory role of ROS and RNS. They also furthered a detailed analysis of redox-sensitive signalling pathways and identification of target molecules, which opened up novel therapeutic perspectives. For this reason, it is advisable to continue analogical studies of RSS which will allow us to fully understand the role of these messengers of redox signalling. For decades it has been known that S-nitrosation of protein–SH groups to SNT is a regulatory me-chanism [3,97], like S-thiolation that leads to generation of mixed disulfides with proteins [98]. In contrast, regulatory function of protein S-sulfhydration yielding protein hydropersulfides (PSSH) began to focus more interest only recently. Nevertheless, the stu-dies have fully documented that the formation of protein hydropersulfides is a fundamental process in cellular thiol-based redox regulation. This conviction is confirmed by a still increasing number of reports of proteins modified by S-sulfhydration. The significance of this process is also evidenced by the widespreadness and abundance of endogenous GSH and protein hydropersulfides [5].

There are many functional similarities and interconnections between NO and H_2_S. It is known that H_2_S and NO mutually affect their concentrations [99]. Since hydropersulfides can be also created in reactions of SNT with HS^−^ anion, thus, the ge-neration of protein SNT is an intermediate step leading finally to more stable hydropersulfides occurring in cells at higher concentrations. It indicates that S-nitrosation can play the role of a driving force of the S-sulfhydration reaction and both these oxi-dative reactions can modify the same cysteine residues in proteins. NO reduces blood pressure via cGMP whereas vasorelaxant action of H_2_S (or RSS) is executed via S-sulfhydration of K_ATP_ channels. Intermolecular S-transnitrosylation reactions consist in NO migration between–SH groups of different molecules although transsulfhydration involves an analogical migration of sulfane sulfur. The formation and accumulation of GSSH can serve to transfer sulfane sulfur to the preferred acceptor protein–SH groups (with a low p*K*_a_ value) in S-sulfhydration reactions. Another similarity between S-sulfhydration and S-nitrosation reactions in mammalian cells is that on the one hand, there are three NOS isoenzymes responsible for NO biosynthesis and on the other, there are three main enzymes responsible for biogenesis of H_2_S and hydropersulfides, i.e. CSE, CBS and MST. All these enzymes differ in tissue distribution and substrate specificity and all produce hydropersulfides facilitating then S-sulfhydration of protein and non-protein thiols. S-sulfhydration, like S-nitrosation, is a reversible process via the Trx system. Thus, the next similarity is occurrence of denitrosation reaction (e.g. S-nitrosoglutathione (GSNO)) and desulfhydration reaction (e.g. GSSH). The difference is that the SNT concentration in tissues and body fluids is low whereas the level of hydropersulfides is much higher [5]. Thus, S-sulfhydration seems to be a more common regulatory me-chanism than S-nitrosation. Both these processes can be compared with the phosphorylation/dephosphorylation regulatory process.

## FUTURE DIRECTIONS AND PROSPECTS

Although the current knowledge of the role of S-sulfhydration process in the regulatory mechanisms constantly expands, there are still many problems which await explanation. First of all, it is necessary to explain whether RSS or H_2_S (or maybe both) are the actual signalling molecules. It should be emphasized that it is more and more often suggested that the sulfane sulfur-containing compounds, and not H_2_S, are real mediators of S-sulfhydration-based signalling. Hence, future studies should make a distinction between biological effects caused by H_2_S and by sulfane sulfur, and the search for new exogenous, safe sulfane sulfur and H_2_S donors with pharmacological potential should be continued.

Moreover, it would also be interesting to elucidate to what extent the biological effect can be attributed to HS^−^ anion and to what extent to H_2_S. H_2_S is known to easily cross cell membrane [100] although it was considered to be impermeable for HS^−^ anion. However, the recent report showed a HS^−^-permeable channel in bacterial cells [101]. Then, studies of Jennings using the human erythrocyte membrane revealed HS^−^ influx in exchange for Cl^−^, catalysed by the anion exchange protein AE1. These results showed that H_2_S and HS^−^ can mediate the transport of acid equivalents across the biological membrane analogically to the cycle for Cl^−^, HCO_3_^−^ and CO_2_ [102].

Secondly, the identification of target molecules able to undergo S-sulfhydration should be continued, which may pave the way for novel prophylactic and therapeutic interventions. Some problems related to specificity of S-sulfhydration reactions, vital in signalling processes, also requires further explanation. Specificity studies require precise estimations of the levels of individual RSS in different subcellular compartments. Thus, development of more precise, sensitive and easy-to-use methods for deter-mination of RSS would be essential for the measurement of their local levels in specific locations within the cell.

The next problem awaiting full elucidation is related to the interaction between H_2_S and NO, and maybe also with CO. All available evidences indicate that these three gasotransmitters cannot be treated as separate molecules but as cooperating factors involved in physiological function of cells in health and disease [103,104]. Therefore, further studies should explain how the three gasotransmitters co-operate to influence the biosynthesis and activity of different reactive species.

Nonetheless, it is worth underlining that the last decade has witnessed a tremendous advance in our knowledge of the rele-vance of S-sulfhydration reaction [2,4,5]. It has been evidenced that this process elicits an extraordinarily widespread influence on different biochemical events and biological processes. Therefore, it can be expected that the next decade will bring a better understanding of the intriguing world of chemistry and bioche-mistry of RSS. There are all indications that further progress in this field and new discoveries and new research methods already can be seen on the horizon.

Most recently, Mishanina et al. in their excellent work proposed a new, interesting concept of RSS biogenesis. They suggested that H_2_S oxidation pathway, considered mainly as a way for disposing the toxic H_2_S excess, should be regarded as the generation route for RSS that could modify target proteins [8]. In fact, as mentioned earlier, mainly hydropersulfides of proteins (SQRSSH) or low-molecular thiols (GSSH) are formed during H_2_S oxidation. GSSH can transfer its sulfane sulfur to target protein as shown in Figure 3. Moreover, thiosulfate, the final product of H_2_S oxidation, is a stable compound containing sulfane sulfur which can transfer this reactive sulfur on to other–SH groups in Rhd-catalysed reaction. Sulfite produced during H_2_S oxidation is also proposed to be RSS due to the possibility of sulfite radical anion (SO^ − ·^_3_) formation. Sulfite oxidation to sulfate or its utilization by Rhd can be the way of limiting the damaging potential of sulfite. Authors compared H_2_S oxidation pathway to the mitochondrial electron transfer, underlining that both are the source of reactive species, namely RSS and ROS, respectively. Moreover, H_2_S oxidation taking place in erythrocytes catalysed by methaemoglobin (Figure 4) also yields RSS (thiosulfate and metal-bound hydropersulfide) [74]. So, this very interesting hypothesis gives a new look at the importance of H_2_S oxidation pathway and RSS signalling potential.

## Abbreviations

CAT: cysteine aminotransferase
CBS: cystathionine-β-synthase
CDO: cysteine dioxygenase
CSE: cystathionine γ-lyase
DADS: diallyl disulfide
DAO: D-amino acid oxidase
DATS: diallyl trisulfide
DHLA: dihydrolipoic acid
ETHE1: persulfide dioxygenase
GAPDH: glyceraldehyde-3-phosphate dehydrogenase
GR: glutathione reductase
GSH: glutathione
MST: 3-mercaptopyruvate sulfurtransferase
PLP: pyridoxal phosphate
PTEN: lipid phosphatase
PTP1B: protein tyrosine phosphatase
Rhd: rhodanese
RNS: reactive nitrogen species
ROS: reactive oxygen species
RSS: reactive sulfur species
RSSH: hydropersulfides
SNT: S-nitrosothiols
SO: sulfite oxidase
SQR: sulfide quinone oxidoreductase
SUR: sulfonylurea receptor
TNFα: tumour necrosis factor alpha
Trx: thioredoxin

## References

[1] Zhang D.X., Gutterman D.D. (2007). Mitochondrial reactive oxygen species-mediated signaling in endothelial cells. Am. J. Physiol. Heart. Circ. Physiol..

[2] Forman H.J., Maiorino M., Ursini F. (2010). Signaling functions of reactive oxygen species. Biochemistry.

[3] Stamler J.S. (1995). S-nitrosothiols and the bioregulatory actions of nitrogen oxides through reactions with thiol groups. Curr. Top. Microbiol. Immunol..

[4] Paulsen C.E., Carroll K.S. (2013). Cysteine-mediated redox signaling: chemistry, biology, and tools for discovery. Chem. Rev..

[5] Ida T., Sawa T., Ihara H., Tsuchiya Y., Watanabe Y., Kumagai Y., Suematsu M., Motohashi H., Fujii S., Matsunaga T. (2014). Reactive cysteine persulfides and S-polythiolation regulate oxidative stress and redox signaling. Proc. Natl. Acad. Sci. U.S.A..

[6] Giles G.I., Tasker K.M., Jacob C. (2001). Hypothesis: the role of reactive sulfur species in oxidative stress. Free Radic. Biol. Med..

[7] Gruhlke M.C., Slusarenko A.J. (2012). The biology of reactive sulfur species (RSS). Plant Physiol. Biochem..

[8] Mishanina T.V., Libiad M., Banerjee R. (2015). Biogenesis of reactive sulfur species for signaling by hydrogen sulfide oxidation pathways. Nat. Chem. Biol..

[9] Stipanuk M.H., Ueki I. (2011). Dealing with methionine/homocysteine sulfur: cysteine metabolism to taurine and inorganic sulfur. J. Inherit. Metab. Dis..

[10] Toohey J.I. (1989). Sulphane sulphur in biological systems: a possible regulatory role. Biochem. J..

[11] Iciek M., Włodek L. (2001). Biosynthesis and biological properties of compounds containing highly reactive, reduced sulfane sulfur. Pol. J. Pharmacol..

[12] Everett S.A., Wardman P. (1995). Perthiols as antioxidants: radical-scavenging and prooxidative mechanisms. Methods Enzymol..

[13] Munday R. (2012). Harmful and beneficial effects of organic monosulfides, disulfides, and polysulfides in animals and humans. Chem. Res. Toxicol..

[14] Shibuya N., Kimura H. (2013). Production of hydrogen sulfide from D-cysteine and its therapeutic potential. Front. Endocrinol. (Lausanne).

[15] Shibuya N., Tanaka M., Yoshida M., Ogasawara Y., Togawa T., Ishii K., Kimura H. (2009). 3-Mercaptopyruvate sulfurtransferase produces hydrogen sulfide and bound sulfane sulfur in the brain. Antioxid. Redox Signal..

[16] Kimura Y., Toyofuku Y., Koike S., Shibua N., Nagahara N., Lefer D., Ogasawara Y., Kimura H. (2015). Identification of H2S3 and H2S produced by 3-mercaptopyruvate sulfurtransferase in the brain. Sci. Rep..

[17] Hylin J., Wood J. (1959). Enzymatic formation of polysulfides from mercaptopyruvate. J. Biol. Chem..

[18] Cavallini D., De Marco C., Mondovi B., Mori B. (1960). The cleavage of cystine by cystathionase and the transulfuration of hypotaurine. Enzymologia.

[19] Szczepkowski T.W., Wood J.L. (1967). The cystathionase-rhodanese system. Biochim. Biophys. Acta.

[20] Abe K., Kimura H. (1996). The possible role of hydrogen sulfide as an endogenous neuromodulator. J. Neurosci..

[21] Łowicka E., Bełtowski J. (2007). Hydrogen sulfide (H_2_S)–the third gas of interest for pharmacologists. Pharmacol. Rep..

[22] Wang R. (2012). Physiological implications of hydrogen sulfide: a whiff exploration that blossomed. Physiol. Rev..

[23] Guo W., Cheng Z.Y., Zhu Y.Z. (2013). Hydrogen sulfide and translational medicine. Acta Pharmacol. Sin..

[24] Kimura Y., Kimura H. (2004). Hydrogen sulfide protects neurons from oxidative stress. FASEB J..

[25] Mikami Y., Shibuya N., Kimura Y., Nagahara N., Ogasawara Y., Kimura H. (2011). Thioredoxin and dihydrolipoic acid are required for 3-mercaptopyruvate sulfurtransferase to produce hydrogen sulfide. Biochem. J..

[26] Bhatia L.L., Zhu Y.C., Ramnath R.D., Wang Z.J., Ankar F.B.M., Whiteman M., Salto-Tellez M., Moore P.K. (2005). Hydrogen sulfide is a novel mediator of lipopolysaccharide-induced inflammation in the mouse. FASEB J..

[27] Whiteman M., Haigh R., Tarr J.M., Gording K.M., Shore A.C., Winyad P.G. (2010). Detection of hydrogen sulfide in plasma and knee-joint synovial fluid from rheumatoid arthritis patients: relation to clinical and laboratory measures of inflammation. Ann. N.Y. Acad. Sci..

[28] Furne J., Saeed A., Levitt M.D. (2008). Whole tissue hydrogen sulfide concentrations are orders of magnitude lower than presently accepted values. Am. J. Physiol. Regul. Integr. Comp. Physiol..

[29] Nagy P. (2015). Mechanistic chemical perspective of hydrogen sulfide signaling. Methods Enzymol..

[30] Nagy P., Palinkas Z., Nagy A., Budai B., Toth I., Vasas A. (2014). Chemical aspects of hydrogen sulfide measurements in physiological samples. Biochim. Biophys. Acta.

[31] Zhang L., Li S., Hong M., Xu Y., Wang S., Liu Y., Qian Y., Zhao J. (2014). A colorimetric and ratiometric fluorescent probe for the imaging of endogenous hydrogen sulphide in living cells and sulphide determination in mouse hippocampus. Org. Biomol. Chem..

[32] Shen X., Peter E., Bir S., Wang R., Kevil C. (2012). Analytical measurement of discrete sulfide pools in biological specimens. Free Radic. Biol. Med..

[33] Kimura Y., Mikami Y., Osumi K., Tsugane M., Oka J., Kimura H. (2013). Polysulfides are possible H_2_S-derived signaling molecules in rat brain. FASEB J..

[34] Toohey J.I. (2011). Sulfur signaling: is the agent sulfide or sulfane?. Anal. Biochem..

[35] Murphy M.P. (2012). Mitochondrial thiols in antioxidant protection and redox signaling: distinct roles for glutathionylation and other thiol modifications. Antioxid. Redox Signal..

[36] Jones D.P., Go Y.M. (2011). Mapping the cysteine proteome: analysis of redox-sensing thiols. Curr. Opin. Chem. Biol..

[37] Winterbourn C.C., Hampton M.B. (2008). Thiol chemistry and specificity in redox signaling. Free Radic. Biol. Med..

[38] Winterbourn C.C. (2015). Are free radicals involved in thiol-based redox signaling?. Free Radic. Biol. Med..

[39] Poole L.B. (2015). The basics of thiols and cysteines in redox biology and chemistry. Free Radic. Biol. Med..

[40] Ferrer-Sueta G., Manta B., Botti H., Radi R., Trujillo M., Denicola A. (2011). Factors affecting protein thiol reactivity and specifity in peroxide reduction. Chem. Res. Toxicol..

[41] Jones D.P. (2008). Radical-free biology of oxidative stress. Am. J. Physiol. Cell Physiol..

[42] Kemp M., Go Y.M., Jones D.P. (2008). Nonequilibrium thermodynamics of thiol/disulfide redox systems: a perspective on redox systems biology. Free Radic. Biol. Med..

[43] Paul B.D., Snyder S.H. (2012). H_2_S signalling through protein sulfhydration and beyond. Nat. Rev. Mol. Cell Biol..

[44] Mustafa A.K., Gadalla M.M., Sen N., Kim S., Mu W., Gazi S.K., Barrow R.K., Yang G., Wang R., Snyder S.H. (2009). H_2_S signals through protein S-sulfhydration. Sci. Signal..

[45] Ono K., Akaike T., Sawa T., Kumagai Y., Wink D.A., Tantillo D.J., Hobbs A.J., Nagy P., Xian M., Lin J., Fukuto J.M. (2014). Redox chemistry and chemical biology of H_2_S, hydropersulfides, and derived species: implications of their possible biological activity and utility. Free Radic. Biol. Med..

[46] Kabil O., Motl N., Banerjee R. (2014). H_2_S and its role in redox signaling. Biochim. Biophys. Acta.

[47] Pan J., Carroll K.S. (2013). Persulfide reactivity in the detection of protein S-sulfhydration. ACS Chem. Biol..

[48] Mueller E.G. (2006). Trafficking in persulfides: delivering sulfur in biosynthetic pathways. Nat. Chem. Biol..

[49] Benson S.W. (1978). Thermochemistry and kinetics of sulfur-containing molecules and radicals. Chem. Rev..

[50] Toohey J.I., Cooper A.J. (2014). Thiosulfoxide (sulfane) sulfur: new chemistry and new regulatory roles in biology. Molecules.

[51] Wood J.L. (1987). Sulfane sulfur. Methods Enzymol..

[52] Kimura H. (2015). Signaling of hydrogen sulfide and polysulfides. Antioxid. Redox. Signal..

[53] Greiner R., Pálinkás Z., Bäsell K., Becher D., Antelmann H., Nagy P., Dick T.P. (2013). Polysulfides link H_2_S to protein thiol oxidation. Antioxid. Redox. Signal..

[54] Francoleon N.E., Carrington S.J., Fukuto J.M. (2011). The reaction of H_2_S with oxidized thiols: generation of persulfides and implications to H_2_S biology. Arch. Biochem. Biophys..

[55] Predmore B.L., Lefer D.J., Gojon G. (2012). Hydrogen sulfide in biochemistry and medicine. Antioxid. Redox Signal..

[56] Vasas A., Dóka É., Fábián I., Nagy P. (2015). Kinetic and thermodynamic studies on the disulfide-bond reducing potential of hydrogen sulfide. Nitric Oxide.

[57] Cuevasanta E., Lange M., Bonanata J., Coitino E.L., Ferrer-Sueta G., Filipovic M.R., Alvarez B. (2015). Reaction of hydrogen sulfide with disulfide and sulfenic acid to form the strongly nucleophilic persulfide. J. Biol. Chem..

[58] Nagy P., Winterbourn C.C. (2010). Rapid reaction of hydrogen sulfide with the neutrophil oxidant hypochlorous acid to generate polysulfides. Chem. Res. Toxicol..

[59] Kimura H. (2012). Metabolic turnover of hydrogen sulfide. Front. Physiol..

[60] Libiad M., Yadav P.K., Vitvitsky V., Martinov M., Banerjee R. (2014). Organization of the human mitochondrial hydrogen sulfide oxidation pathway. J. Biol. Chem..

[61] Hildebrandt T.M., Grieshaber M.K. (2008). Three enzymatic activities catalyze the oxidation of sulfide to thiosulfate in mammalian and invertebrate mitochondria. FEBS J..

[62] Jackson M.R., Melideo S.L., Jorns M.S. (2012). Human sulfide: quinone oxidoreductase catalyzes the first step in hydrogen sulfide metabolism and produces a sulfane sulfur metabolite. Biochemistry.

[63] Cherney M.M., Zhang Y., James M.N., Weiner J.H. (2012). Structure-activity characterization of sulfide:quinone oxidoreductase variants. J. Struct. Biol..

[64] Goubern M., Andriamihaja M., Nübel T., Blachier F., Bouillaud F. (2007). Sulfide, the first inorganic substrate for human cells. FASEB J..

[65] Kabil O., Banerjee R. (2014). Enzymology of H_2_S biogenesis, decay and signaling. Antioxid. Redox Signal..

[66] Kabil O., Banerjee R. (2012). Characterization of patient mutations in human persulfide dioxygenase (ETHE1) involved in H_2_S catabolism. J. Biol. Chem..

[67] Hildebrandt T.M., Di Meo I., Zeviani M., Viscomi C., Braun H.P. (2013). Proteome adaptations in Ethe1-deficient mice indicate a role in lipid catabolism and cytoskeleton organization via post-translational protein modifications. Biosci. Rep..

[68] Tiranti V., Viscomi C., Hildebrandt T., Di Meo I., Mineri R., Tiveron C., Levitt M.D., Prelle A., Fagiolari G., Rimoldi M., Zeviani M. (2009). Loss of ETHE1, a mitochondrial dioxygenase, causes fatal sulfide toxicity in ethylmalonic encephalopathy. Nat. Med..

[69] Sörbo B.H. (1955). Rhodanese. Met. Enzymol..

[70] Melideo S.L., Jackson M.R., Johns M.S. (2014). Biosynthesis of a central intermediate in hydrogen sulfide metabolism by a novel human sulfurtransferase and its yeast ortholog. Biochemistry.

[71] Koj A., Frendo J. (1967). Oxidation of thiosulphate to sulphate in animal tissues. Folia Biol. (Krakow).

[72] Koj A., Frendo J., Janik Z. (1967). [^35^S]Thiosulphate oxidation by rat liver mitochondria in the presence of glutathione. Biochem. J..

[73] Skarżyński B., Szczepkowski T.W., Weber M. (1960). Investigations on the oxidation of thiosulphate in the animal organism. Acta Biochim. Pol..

[74] Vitvitsky V., Yadav P.K., Kurthen A., Banerjee R. (2015). Sulfide oxidation by a noncanonical pathway in red blood cells generates thiosulfate and polysulfides. J. Biol. Chem..

[75] Westley A.M., Westley J. (1991). Biological sulfane sulfur. Anal. Biochem..

[76] Ogasawara Y., Soda S., Tanabe S. (1994). Tissue and subcellular distribution of bound and acid-labile sulfur, and the enzymic capacity for sulfide production in the rat. Biol. Pharm. Bull..

[77] Krishnan N., Fu C., Pappin D.J., Tonks N.K. (2011). H_2_S-induced sulfhydration of the phosphatase PTP1B and its role in the endoplasmic reticulum stress response. Sci. Signal..

[78] Kim E.J., Feng J., Bramlett M.R., Lindahl P.A. (2004). Evidence for a proton transfer network and a required persulfide-bond-forming cysteine residue in Ni-containing carbon monoxide dehydrogenases. Biochemistry.

[79] De Beus M.D., Chung J., Colón W. (2004). Modification of cysteine 111 in Cu/Zn superoxide dismutase results in altered spectroscopic and biophysical properties. Protein Sci..

[80] Finazzi-Agrò A., Mavelli I., Cannella C., Federici G. (1976). Activation of porcine heart mitochondrial malate dehydrogenase by zero valence sulfur and rhodanese. Biochem. Biophys. Res. Commun..

[81] Sandy J.D., Davies R.C., Neuberger A. (1975). Control of 5 aminolaevulinate synthetase activity in rhodopseudomonas spheroides. A role for trisulphides. Biochem. J..

[82] Massey V., Edmondson D. (1970). On the mechanism of inactivation of xanthine oxidase by cyanide. J. Biol. Chem..

[83] Branzoli U., Massey V. (1974). Evidence for an active site persulfide residue in rabbit liver aldehyde oxidase. J. Biol. Chem..

[84] Ohno K., Okuda K., Uehara T. (2015). Endogenous S-sulfhydration of PTEN helps protect against modification by nitric oxide. Biochem. Biophys. Res. Commun..

[85] Craig T.J., Ashcroft F.M., Proks P. (2008). How ATP inhibits the open K(ATP) channel. J. Gen. Physiol..

[86] Peers C., Bauer C.C., Boyle J.P., Scragg J.L., Dallas M.L. (2012). Modulation of ion channels by hydrogen sulfide. Antioxid. Redox Signal..

[87] Mustafa A.K., Sikka G., Gazi S.K., Steppan J., Jung S.M., Bhunia A.K., Barodka V.M, Gazi F.K., Barrow R.K., Wang R. (2011). Hydrogen sulfide as endothelium-derived hyperpolarizing factor sulfhydrates potassium channels. Circ. Res..

[88] Gade A.R., Kang M., Akbarali H.I. (2013). Hydrogen sulfide as an allosteric modulator of ATP-sensitive potassium channels in colonic inflammation. Mol. Pharmacol..

[89] Kang M., Hashimoto A., Gade A., Akbarali H.I. (2015). Interaction between hydrogen sulfide-induced sulfhydration and tyrosine nitration in the KATP channel complex. Am. J. Physiol. Gastrointest. Liver Physiol..

[90] Yang G., Wu L., Jiang B., Yang W., Qi J., Cao K., Meng Q., Mustafa A.K., Mu W., Zhang S. (2008). H_2_S as a physiologic vasorelaxant: hypertension in mice with deletion of cystathionine γ-lyase. Science.

[91] Sen N., Paul B.D., Gadalla M.M., Mustafa A.K., Sen T., Xu R., Kim S., Snyder S.H. (2012). Hydrogen sulfide-linked sulfhydration of NF-κB mediates its antiapoptotic actions. Mol. Cell.

[92] Zhen Y., Pan W., Hu F., Wu H., Feng J., Zhang Y., Chen J. (2015). Exogenous hydrogen sulfide exerts proliferation/anti-apoptosis/angiogenesis/migration effects via amplifying the activation of NF-κB pathway in PLC/PRF/5 hepatoma cells. Int. J. Oncol..

[93] Banerjee R., Maulik S.K. (2002). Effect of garlic on cardiovascular disorders: a review. Nutr. J..

[94] Schäfer G., Kaschula C.H. (2014). The immunomodulation and anti-inflammatory effects of garlic organosulfur compounds in cancer chemoprevention. Anticancer Agents Med. Chem..

[95] Iciek M., Kwiecień I., Włodek L. (2009). Biological properties of garlic and garlic-derived organosulfur compounds. Environ. Mol. Mutagen..

[96] Benavides G.A., Squadrito G.L., Mills R.W., Patel H.D., Isbell T.S., Patel R.P., Darley-Usmar V.M., Doeller J.E., Kraus D.W. (2007). Hydrogen sulfide mediates the vasoactivity of garlic. Proc. Natl. Acad. Sci. U.S.A..

[97] Hess D.T., Stamler J.S. (2012). Regulation by S-nitrosylation of protein post-translational modification. J. Biol. Chem..

[98] Włodek L., Iciek M. (2003). Protein S-thiolation as an antioxidative and regulatory mechanism. Postepy Biochem..

[99] Kolluru G.K., Shen X., Kevil C.G. (2013). A tale of two gases: NO and H_2_S, foes or friends for life?. Redox Biol..

[100] Mathai J.C., Missner A., Kügler P., Saparov S.M., Zeidel M.L., Lee J.K., Pohl P. (2009). No facilitator required for membrane transport of hydrogen sulfide. Proc. Natl. Acad. Sci. U.S.A..

[101] Czyżewski B.K., Wang D. (2012). Identification and characterization of a bacterial hydrosulphide ion channel. Nature.

[102] Jennings M.L. (2013). Transport of H_2_S and HS^−^ across the human red blood cell membrane: rapid H_2_S diffusion and AE1-mediated Cl^−^/HS^−^ exchange. Am. J. Cell Physiol..

[103] Lu C., Kavalier A., Lukyanov E., Gross S.S. (2013). S-sulfhydration/desulfhydration and S-nitrosylation/denitrosylation: a common paradigm for gasotransmitter signaling by H_2_S and NO. Methods.

[104] Fukuto J.M., Carrington S.J., Tantillo D.J., Harrison J.G., Ignarro L.J., Freeman B.A., Chen A., Wink D.A. (2012). Small molecule signaling agents: the integrated chemistry and biochemistry of nitrogen oxides, oxides of carbon, dioxygen, hydrogen sulfide, and their derived species. Chem. Res. Toxicol..

